# QATCHEPP: A quality assessment tool for critical health promotion practice

**DOI:** 10.3389/fpubh.2023.1121932

**Published:** 2023-03-21

**Authors:** Lily O'Hara, Jane Taylor

**Affiliations:** ^1^Department of Public Health, College of Health Sciences, QU Health, Qatar University, Doha, Qatar; ^2^School of Health, University of the Sunshine Coast, Maroochydore, QLD, Australia

**Keywords:** health promotion, public health, reflective practice, critical systems heuristics, values, principles, quality assessment, quality improvement

## Abstract

**Background:**

The origins of health promotion are based in critical practice; however, health promotion practice is still dominated by selective biomedical and behavioral approaches, which are insufficient to reduce health inequities resulting from the inequitable distribution of structural and systemic privilege and power. The Red Lotus Critical Health Promotion Model (RLCHPM), developed to enhance critical practice, includes values and principles that practitioners can use to critically reflect on health promotion practice. Existing quality assessment tools focus primarily on technical aspects of practice rather than the underpinning values and principles. The aim of this project was to develop a quality assessment tool to support critical reflection using the values and principles of critical health promotion. The purpose of the tool is to support the reorientation of health promotion practice toward a more critical approach.

**Research design:**

We used Critical Systems Heuristics as the theoretical framework to develop the quality assessment tool. First, we refined the values and principles in the RLCHPM, then created critical reflective questions, refined the response categories, and added a scoring system.

**Results:**

The Quality Assessment Tool for Critical Health Promotion Practice (QATCHEPP) includes 10 values and associated principles. Each value is a critical health promotion concept, and its associated principle provides a description of how the value is enacted in professional practice. QATCHEPP includes a set of three reflective questions for each value and associated principle. For each question, users score the practice as strongly, somewhat, or minimally/not at all reflective of critical health promotion practice. A percentage summary score is generated with 85% or above indicative of strongly critical practice, 50% ≤ 84% is somewhat critical practice, and < 50% minimally or does not reflect critical practice.

**Conclusion:**

QATCHEPP provides theory-based heuristic support for practitioners to use critical reflection to assess the extent to which practice aligns with critical health promotion. QATCHEPP can be used as part of the Red Lotus Critical Promotion Model or as an independent quality assessment tool to support the orientation of health promotion toward critical practice. This is essential to ensure that health promotion practice contributes to enhancing health equity.

## 1. Introduction

### 1.1. Critical health promotion practice

Health promotion practice is described in the CompHP Core Competencies Framework for Health Promotion developed by the International Union for Health Promotion and Education (IUHPE) (1). Health promotion practice refers to health promotion programs, projects, policy, strategies, and initiatives. Health promotion practitioners refers to those whose main role is health promotion practice, for example people who work in health promotion specific government, non-government, or community organizations. Health promotion practice is also undertaken as a component of other practitioners' roles, for example clinical and allied health practitioners, educators, urban planners, and climate and social justice activists. The CompHP Core Competencies Framework for Health Promotion was informed by key international health promotion documents, leaders, and practitioners. It is used internationally for the accreditation of health promotion practitioners and university academic programs, the development of health promotion position descriptions, and professional development programs (1).

The CompHP Core Competencies Framework for Health Promotion includes nine domains of competency standards for practice: enable change to reduce health inequities; advocate and build capacity for health and wellbeing; mediate through partnerships to enhance the impact and sustainability of health promotion; communicate appropriately with diverse audiences; demonstrate leadership for health promotion action; conduct community health and wellbeing assessment; plan evidence-based health promotion programs; implement ethical health promotion programs; conduct appropriate evaluation and research to determine efficacy and effectiveness of health promotion programs. The competency standards are underpinned by a set of ethical values required to be enacted in health promotion practice.

The origins of health promotion are based in critical practice (2, 3). However, health promotion practice is still dominated by a selective health promotion approach which adopts a biomedical and behavioral health paradigm and tends to focus on populations that are structurally and systemically privileged. This results in health promotion programs focusing on changing individual level behaviors related to disease rather than the broader structural and systemic determinants of health and wellbeing (4–6). Recognizing the broader determinants of health but developing health promotion programs that focus on individual behaviors has been criticized as “lifestyle drift” (7, 8) or “downstream drift” (6), and using the “lazy language of lifestyles” (9, 10). The selective approach is insufficient to address the full range of health and wellbeing determinants and reduce health inequities (4–6, 11–13).

In contrast to selective health promotion, critical health promotion is “a social justice approach to health promotion that is underpinned by a system of values and related principles that supports the reflective process of explicitly identifying and challenging dominant social structures and discourses that privilege the interests of the powerful and contribute to health and wellbeing inequities” (14). The values of critical health promotion are the practice concepts that are most important in professional practice, for example, health equity, systems science, salutogenesis, and non-maleficence. The principles of critical health promotion are the actions taken to accomplish the values, for example the value of health equity is accomplished by prioritizing working with people and communities that are most impacted by the inequitable distribution of structural and systemic privilege and power (15). However, rarely are the values and principles that underpin health promotion programs made explicit in research and practice environments (16, 17). As such, health promotion activity generally reflects and reinforces the dominant selective approach (11, 18), for which evidence of effectiveness is more plentiful due to its specific focus on behavioral factors. However, as per Nutbeam's inverse evidence law, there is relatively little evidence about the effectiveness of health promotion programs addressing the broader structural and systemic determinants of health and wellbeing and health equity (19). A selective approach is not fully reflective of what is considered good health promotion practice (4, 11). As such, good health promotion practice should be shared with the field to enable maximal adoption (20).

### 1.2. Quality concepts in health promotion

Quality in health promotion practice has been defined as the extent to which key predictors of effect are incorporated in a program (21). Øvretviet (22) proposed that ideas about quality emerging from the quality movement were compatible with good practice in health promotion, and advocated for widespread adoption of such quality concepts in order to improve the quality of health promotion practice. He proposed that a combination of the three dimensions of quality be adopted by health promotion practitioners: consumer quality (level of consumer and community satisfaction), professional quality (quality of program planning and design, including methods for reconciling conflicts between community and professional views and higher-level requirements), and management quality (quality of implementation, efficacy of resource use, meeting higher-level requirements). Øvretviet posited that this approach would provide a balance between the consumer and professional dimensions, rather than privileging the professional and managerial dimensions at the expense of the consumer dimension, give equal status to the process and outcome of health promotion programs, and incorporate considerations of cost and higher-level directives. He believed that incorporating the three domains into quality assessment would also address the requirement for health promotion to work with priority populations to reduce health inequities.

Speller et al. (23) developed quality assurance standards for health promotion practice in the United Kingdom. The authors noted that the standards were limited to the inputs and processes of health promotion activity. Fazal et al. (24) developed criteria to distinguish between worst, promising, and best practices for health promotion based on impact, adaptability, and quality of evidence. These criteria focus specifically on the quality of health promotion “interventions”. The IUHPE competency framework provides the first set of international standards that describe the requirements for the implementation of good quality health promotion (1). Individual countries have also developed competency frameworks, for example Australia (25), Israel (26), and New Zealand (27). Whilst these frameworks provide clear standards or statements describing good quality practice, they are not designed to be used by practitioners as quality assessment tools.

Quality assessment tools are used in the quality improvement process. Quality improvement has multiple definitions all of which involve the common principle of a continuous, systematic process to improve health practices and therefore enhance health outcomes for people and communities (28). In health promotion, quality assessment tools support practitioners to determine the level or extent to which good quality practice is evident in a health promotion program and identify areas for improvement (29). Much of the development of health promotion quality assessment tools has taken place in settings-based health promotion. For example, in the field of workplace health promotion, many quality assessment checklists have been developed such as the United States Centers for Disease Control Worksite Health ScoreCard (30), the HERO Health and Well-Being Best Practices Scorecard (31, 32), and WELCOA's Worksite Health Promotion Benchmarks (33). Other quality assessment tools focus on particular aspects of practice such as program outcomes (34).

At a broader level, the Healthy Austria Fund produced Quality Criteria for Basic Principles of Health Promotion including nine principles with criteria and indicators for each principle. The principles include positive, comprehensive and dynamic concept of health; health equity; resource orientation; empowerment; setting and determinant orientation; target group orientation; participation of the actors in the setting; networking; and sustainability of the changes (35). The Quality Criteria instrument is intended to be used as a guide in the development and assessment of health promotion projects. It does not include a scoring system. Preffi 2.0 is a quality assessment tool that includes 39 indicators of effective health promotion programs grouped into clusters, which are scored as weak, moderate, or strong, depending on the yes/no answers for each indicator in the cluster (36). Each of the clusters focuses on technical aspects of health promotion practice, including contextual conditions, analysis, selection and development of interventions, implementation, and evaluation.

As an outcome of the Getting Evidence into Practice Project funded by the European Commission, the Netherlands Institute for Health Promotion and Illness Prevention and the Flemish Institute for Health Promotion produced the European Quality Instrument for Health Promotion (EQUIHP) for the purpose of improving the quality of health promotion practice (37, 38). EQUIHP includes 95 indicators grouped into 13 criteria for effective health promotion, which are further grouped into four domains: framework of health promotion principles; project development and implementation; project management; and sustainability. The framework of health promotion principles includes indicators for a positive and comprehensive approach to health, attention for the broad determinants of health, participation, empowerment, equity, and equality. Each indicator is framed as one or more questions with three possible responses: no (not achieved), partly (partly achieved), or yes (achieved). The user manual for EQUIHP does not include a quantitative scoring system, however research projects that have used the instrument as a quality assessment tool have devised their own scoring systems (39–41).

The German Cooperation Network “Equity in Health” developed the Criteria for Good Practice in Health Promotion Addressing Social Determinants, which provides a framework to plan and implement health promotion programs to address the social determinants of health (42). The framework includes 12 criteria described as technical concepts: concept and project planning; target group orientation; settings approach; integrating intermediaries; sustainability; low-threshold methodology; participation; empowerment; integrated action/networking; quality management; documentation and evaluation; and capturing cost effectiveness. For each criterion, a definition of the criterion is provided, followed by an explanation and example of different implementation levels across a continuum. The number of levels vary across the criteria between three and six, with the highest level representing good quality practice. The Criteria for Good Practice document is designed to be used as a qualitative reflection tool and does not include a quantitative scoring system.

The health promotion quality assessment tools developed to date focus primarily or exclusively on technical aspects of practice, and most do not have a scoring system that enables quantitative assessment of the extent to which the practice aligns with good or best health promotion practice. In addition, existing tools do not explicitly incorporate health promotion values and their related principles. It is important that practitioners develop the skills to critically reflect on the underlying values and principles of health promotion to enable them to reorient practice toward a more critical approach (43).

### 1.3. Critical reflection

Critical reflection is a professional skill integral to the practice of critical health promotion. It involves examining the underlying assumptions of a health promotion program and the source of such assumptions (43). Through critical reflection, practitioners increase their consciousness about the dominant values and principles of health promotion programs, and the implications for whom they are intended (22–25). Health promotion practitioners are encouraged to engage in critical reflection at individual and team levels as a mechanism for enhancing the quality of practice (44–46). Johnson and MacDougall describe critical reflection as an active process that requires practitioners to:

…describe, question and challenge our assumptions, beliefs, values, and theories about why things happen and explore how things may be different. It behooves us to think critically, seek feedback, and to move out of our comfort zones and individual frame of reference as we question the assumptions on which we base our practice [(46), p. 250–1].

Key elements of critical reflection include questioning underlying assumptions, a social focus as distinct from an individual focus, the analysis of power relations, and emancipation (46). Critical reflection assists practitioners to better understand and learn about their health promotion practice, and to change, enhance or transform their practice in the future (44–49). Practice elements might include the philosophical approach, values and principles, theory and models used (46, 50), all of which underpin and guide the design, implementation, and evaluation of health promotion programs (50). Critical reflection is crucial to improve and transform practice if health promotion is to affect the broader political economic, social, and cultural determinants of health, and thereby enhance health equity (46).

While acknowledged as important, there has been insufficient attention paid to critical reflection as a key health promotion skill (45). As such, there is a lack of awareness about the influence of the perspectives of those that plan and deliver health promotion programs and their underlying societal and professional norms, on the nature of practice (45). Critically reflecting on practice is not always a priority for practitioners due to the focus on technical aspects of their everyday work (47). Practitioners need to allocate time for critical reflection individually, as a team or with a practice mentor (45–48, 51). Critical reflection processes and tools are required to support practitioners (45–48, 51). Existing processes and tools provide some guidance for reflecting on the various components of a health promotion program at a technical level, but few focus on the critical elements of reflective practice. Fleming (45) proposed a typology for enhancing the “neglected art” of reflective practice in health promotion. The typology outlines a series of reflective questions that practitioners can ask individually and at a team level about the context and process of program planning across the components of a health promotion program. However, this typology does not focus specifically on making the values and principles that underpin health promotion programs explicit. Practitioners need models and frameworks to support their engagement in critical reflection. To respond to this need, we developed the Red Lotus Critical Health Promotion Model (RLCHPM), which incorporates the practice of critical reflection.

### 1.4. Red Lotus Critical Health Promotion Model

Document analysis was used to develop the original version which was titled the Red Lotus Health Promotion Model and first published in 2007 (50). The model was underpinned by critical systems theory (52, 53) and used the red lotus plant to symbolize the components of health promotion, including health status (pod), people's characteristics (stamens), environmental determinants of health and wellbeing (first petal layer), community assessment (second petal layer), planning (third petal layer), implementation (fourth petal layer), evaluation (fifth petal layer), values (roots), principles (stems), and sustainability (leaves). The rationale for choosing the red lotus plant as the symbol and its cultural, culinary, medicinal, and spiritual significance is described in detail elsewhere (50). The content of the model was derived from the international health promotion declarations and charters produced by the World Health Organization and existing health promotion models and frameworks.

Most significantly, the model included a system of ethical, philosophical, and technical values and associated principles that characterized a critical approach to health promotion, which had been identified as a major gap in other health promotion models (16, 50). It is important to note that the terms “values” and “principles” are not used interchangeably in the model. The phrase “values and principles” is a succinct expression of the more complete phrase “values and associated principles.” The values in the model refer to the key health promotion concepts that underpin critical practice, and the associated principles refer to the actions required to enact the value. For example, the model includes the value of holistic health paradigm, and the associated principle of framing health as a complex concept that includes physical, mental, spiritual, social, cultural, and environmental aspects of wellbeing. The values and principles were derived from World Health Organization health promotion charters and declarations, the health promotion literature (16), and health promotion competency frameworks at the international (54) and national (25) levels.

Over the following years, the Red Lotus Health Promotion Model was applied in teaching (55, 56), research (16, 43, 52, 57–60) and practice. To assess the impact of the model on our graduates' practice, we conducted a mixed-methods study with our former students who had graduated between 2008 and 2016 using an online survey and semi-structured interviews (55). Most participants were knowledgeable about and confident in using the model, and felt it was relevant and useful to their practice. Using the heuristic to evaluate their own health promotion practice, most participants rated their practice as somewhat or strongly aligned with a critical approach. However, qualitative findings identified the need for more structured support for evaluating the criticality of their own and others' health promotion practice.

We have also engaged in ongoing reflection on the model (53) and gathered informal feedback from students, researchers, and practitioners using the model. As a result of the formal and informal feedback, we identified several potential areas for improvement in the model. We recognized that the process of critical reflection was not represented in the model, and that the values and principles were not at the base of the model. We had categorized values and principles into philosophical, ethical, and technical domains and users of the model interpreted this categorization to mean that not all values and principles were important to ethical practice. Some of the model's 19 values and principles were difficult for users to translate into practice due to their complexity. We also identified that the values and principles did not fully explicate the structural and systemic underpinnings of critical health promotion. Furthermore, the term “critical” was missing from the title of the model, thereby the critical intent of the model was not explicit in the title. Finally, we identified that the model needed updating to reflect current developments in understanding about the breadth and depth of intersecting structural and systemic determinants of health and wellbeing.

**Figure 1:**
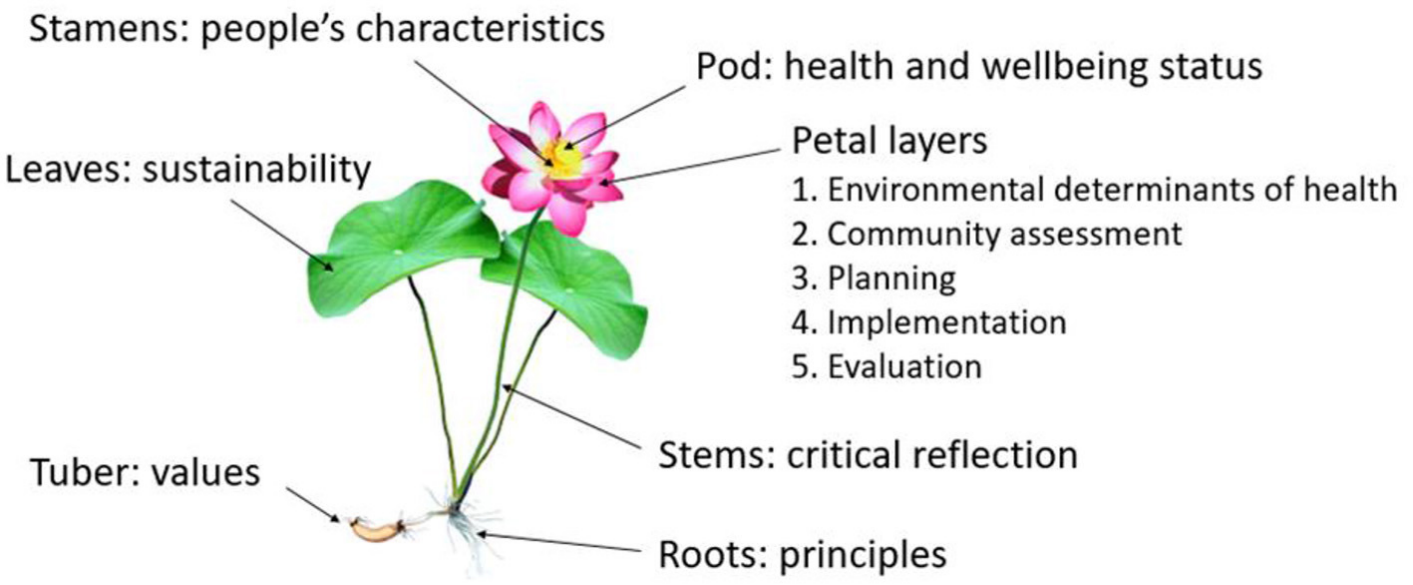
Red Lotus Critical Health Promotion Model. Reproduced with permission from Springer Nature (15).

Version 2 was published as the Red Lotus Critical Health Promotion Model (RLCHPM) in 2021 (14) and 2022 (52) (Figure 1) (59). The tuber and roots have been reassigned to represent the values and principles, and the stems to represent the critical reflection process. The tuber and roots are the foundation of the plant which more appropriately represent the values and principles underpinning critical health promotion. The stems connect the tuber and roots to the flower and leaves, which more appropriately represent the role of critical reflection in applying the values and principles in practice. The number of values and principles has been reduced to 10 without categorization into domains, with some refinement of the wording of the values and principles to reflect the structural and systemic underpinnings of critical health promotion, and emergent determinants of health and wellbeing are included. The RLCHPM has been used in our teaching including as a framework for examining the determinants of health and wellbeing, identifying priority populations, and the development of strategies to address priority issues. We have also used the RLCHPM in research (61, 62), and further studies are required to test its effectiveness in practice.

The values and principles system included in the RLCHPM has been used as a heuristic to evaluate the extent to which health promotion practice aligns with a critical health promotion approach (15, 55). For example, O'Hara et al. (57) used the heuristic to evaluate weight related public health initiatives spanning a 10 year period in Australia. Based on a critical discourse analysis of the initiatives' documentation, they rated the program as strongly critical, somewhat critical, somewhat selective, or strongly selective for each value and associated principle. They found that although there was some evidence of a somewhat critical approach, overall, these initiatives were strongly aligned with a selective approach. In addition to applying the heuristic in research projects, we have used the heuristic in our undergraduate and graduate courses as an assessment task, whereby students critique an existing health promotion program and evaluate the extent to which the program aligns with a critical approach. To support this critique, we ask reflective questions to help students identify the range of evidence that may demonstrate the implicit or explicit application of the values and principles in the program being evaluated. Over the years of doing this, it has become strongly apparent to us that these *ad hoc* reflective questions are key to enabling the critical reflection process. As such we concluded that structuring the reflective questions into a formalized tool would significantly enhance the critical reflection process.

Through the application of the heuristic in our own research, together with student and graduate feedback, and our own ongoing reflection, the need for a quality assessment tool to support critical reflection on the alignment of health promotion practice with a critical health promotion approach became apparent.

## 2. Research design

### 2.1. Aim

The aim of this project was to develop a quality assessment tool to support critical reflection on health promotion practice.

### 2.2. Epistemology and approach

The research was guided by constructionist epistemology, which acknowledges that the knowledge generated was constructed by us, and informed by our experiences within our professional and personal contexts (63). We both come from health promotion practice backgrounds and have worked in various positions as health promotion practitioners in government, non-government, and community organizations prior to working in academia. We therefore approach this research as both practitioners and researchers. As such, the research was also guided by a pragmatic approach (64), whereby the knowledge generated is intended to be applied in a practice environment. In this context, the intended application is the reorientation of health promotion programs away from the more dominant selective approach, and toward the more effective critical approach.

### 2.3. Theoretical framework

Critical Systems Heuristics (CSH) was used as the theoretical framework to develop the quality assessment tool. CSH is a framework for reflective practice which requires the development of the critical competence of practitioners to engage in systems thinking and reflective discourse to identify the dominant values of a system (65–67). The term heuristic derives from the Greek term heurisko which means to assist to discover (29). In this context, the term heuristic refers to a practical tool to assist practitioners and the communities they work with to engage in critical reflective dialogue about the boundary judgements implicitly or explicitly influencing a health promotion program (25). CSH assists practitioners to make sense of the broader context of a health promotion program by asking purposeful questions to identify the sources of knowledge, power, motivation, and legitimation for those involved in and those affected by the health promotion program (68). Through this questioning process, the assumptions or judgements that multiple program stakeholders unconsciously or consciously hold about the program are made explicit. These prior (apriori) judgements are referred to as boundary judgements which are explored through the process of boundary critique. In this project, boundary critique involves a structured critical dialogue using a heuristic of critical questions (66–69) that focus on *what ought to be* present in a critical health promotion program and *what is* present in a current or proposed health promotion program.

Some boundary judgements are more privileged than others due to structural power imbalances. These privileged boundary judgements are referred to as normative content, which constitutes the accepted and unchallenged value judgements of those who hold the power and excludes those that live with the experience and implications of such value judgements in a health promotion program (69). The value judgements of health promotion practitioners are legitimized through their professional roles and therefore influence the community assessment, planning, implementation, and evaluation components of a health promotion program, but practitioners are not necessarily accountable to the communities they work with. CSH enables practitioners to engage in boundary critique by using a heuristic tool to support reflective practice with the range of stakeholders throughout all components of a health promotion program (68, 69). The outcome of this critical reflection is intended to provide guidance for practitioners in the design of new programs or the reorientation of existing programs toward a more critical health promotion approach.

### 2.4. Method

The method used to develop the quality assessment tool consisted of first, refining the 10 values and principles in the Red Lotus Critical Health Promotion Model. Second, we created critical reflective questions based on the content of each value and principle and the framework of CSH boundary judgement (68). Third, we changed the categorization in the heuristic from strongly or somewhat reflective of critical or selective health promotion to strongly critical, somewhat critical, minimally critical/not at all critical, and added a new category for no evidence available. We removed the categories related to selective health promotion because the focus of the tool is on critical health promotion. Fourth, a numerical score was allocated to each category to allow for the quantitative assessment of each question, each value and principle, and the program overall. A scoring calculation method was developed to allow users to interpret the results. Fifth, we included the requirement to provide evidence (if available) from the program source to support the assigned ratings.

To pilot test QATCHEPP, one of us (LOH) trialed it as part of an assignment for graduate public health students in 2022. Students were required to critically review a health promotion program using QATCHEPP. All nine students in the course were health practitioners, four of whom were health promotion practitioners, two of whom used the assignment to assess the health promotion program they were currently working on in their professional roles. At the conclusion of the course, as part of the continuous quality improvement process, students were asked to provide feedback on the clarity of the questions in the tool, the ease of use, and suggestions for amendments. All students found the tool relatively easy to use, however, they found some reflective questions somewhat unclear, and some aspects of the scoring system not straightforward or intuitive. As a result of suggestions from the students, we made several amendments to the wording of questions and the scoring system.

## 3. Results

The **Q**uality **A**ssessment **T**ool for **C**ritical **He**alth **P**romotion **P**ractice (QATCHEPP—pronounced catchep) includes 10 values and associated principles (Table 1). The values that characterize critical health promotion practice are: priority populations determined by structural inequality; holistic health paradigm; salutogenic approach; systems science; assume people do the best they can for their wellbeing; work with people as an ally; empowering engagement processes; comprehensive use of evidence, theory, and models; maximum beneficence; and non-maleficence is a priority consideration. To assist practitioners to identify how critical health promotion is distinct from selective health promotion practice, QATCHEPP also notes *in italics* the values and principles associated with selective health promotion practice. QATCHEPP includes a set of three reflective questions for each of the 10 values and associated principles to guide the practitioner's critical reflection on a health promotion program. In QATCHEPP, the term program encompasses all types of health promotion action including a project, policy, strategy, or initiative. QATCHEPP reflective questions are theoretically informed and designed to assist practitioners to interrogate the key features of each value and principle and make an evaluative judgement about the extent to which they are enacted in practice in the health promotion program. The set of three questions for each value and associated principle follows a sequential order intended to guide the practitioner through a stepped process of identifying relevant evidence from the program outputs. Outputs may include program plans, evaluation reports, websites, journal articles, conference posters and presentations, community presentations, program briefs, funding applications, program communications, media releases and posts, program resources, meeting minutes, or any other outputs from the program.

**Table 1:** Quality assessment tool for critical health promotion practice.

**Number**	**Critical and selective values**	**Critical and selective principles**	**Critical reflection questions**	**Strongly critical 2**	**Somewhat critical 1**	**Minimally or not at all critical 0**	**No evidence available 0**	**Evidence from source to support the rating**	**Total score for each value**
1	Critical: Priority population determined by structural inequality	Critical: In recognition that the enjoyment of the highest attainable standard of health is a fundamental human right, prioritizing working with people and communities that are most impacted by the inequitable distribution of structural and systemic privilege and power	1a Were criteria used to identify the priority population related to the inequitable distribution of structural and systemic privilege and power?						
			1b Is the priority population described as a priority population as opposed to being labeled as a disadvantaged, marginalized, vulnerable, at risk, or target population?						
	*Selective: Priority populations determined by structural privilege*	*Selective: Without explicit recognition that the enjoyment of the highest attainable standard of health is a fundamental human right, prioritizing working with people and communities that benefit from structural and systemic privilege and power*	1c Does the program use discourse that reflects social and environmental justice ideologies as opposed to neoliberal, capitalist, and colonialist ideologies?						
2	Critical: Holistic health paradigm	Critical: Framing health as a complex concept that includes physical, mental, spiritual, social, cultural, and environmental aspects of wellbeing	2a Does the program address multiple holistic health and wellbeing aspects?						
			2b Does the program describe the connections between the different aspects of health and wellbeing?						
	*Selective: Biomedical-behavioral health paradigm*	*Selective: Framing health as the absence of disease or injury, and the absence of “unhealthy” behaviors, primarily related to the body and mind, excluding social, spiritual, and environmental health and wellbeing*	2c Does the program evaluation focus on multiple holistic health and wellbeing outcomes?						
3	Critical: Salutogenic approach	Critical: Enhancing strengths and assets that create and support health, wellbeing, resilience, sense of coherence, happiness, self-respect, and meaning in life, in addition to structural and systemic factors that create poor health and wellbeing	3a Does the program focus on the creation of good health and wellbeing as the primary outcome?						
			3b Does the program present health and wellbeing related data about people and environments from a salutogenic perspective?						
	*Selective: Deficit approach*	*Selective: Focusing on needs, including deficits, problems, or “risk factors” for disease and injury using prevention/preventive strategies*	3c Does the program consistently use salutogenic discourse?						
4	Critical: Systems science	Critical: Using systems science, which recognizes that the determinants of health and wellbeing operate in multiple complex intersecting ecosystems (from the individual to the family, group, community, population, and global level), which need to be addressed to achieve sustainable health and wellbeing outcomes	4a Does the program refer to systems science and/or systems thinking as the underpinning framework?						
			4b Does the program identify and address intersecting intrapersonal, interpersonal, and environmental determinants of health and wellbeing at multiple levels?						
	*Selective: Reductionist science*	*Selective: Using reductionist science, which assumes that health outcomes are caused by discrete “risk factors” for disease and injury and does not address the full range of intersecting determinants of health and wellbeing that operate across multiple levels*	4c Does the program evaluation focus on health and wellbeing outcomes at multiple levels?						
5	Critical: Assume that people are doing their best for their wellbeing	Critical: Assuming that when left to their own devices, people will do the best for their wellbeing including that of their families, communities, and environment, given their circumstances and available resources	5a Does the program focus on improving circumstances and resources that support health and wellbeing?						
			5b Does the program implicitly or explicitly blame people for their circumstances, available resources, and consequential poor health and wellbeing outcomes?						
	*Selective: Assume that people are not doing the best for their wellbeing*	*Selective: Assuming that when left to their own devices, people will naturally adopt “unhealthy lifestyles” and harmful environmental behaviors, irrespective of their circumstances and available resources*							
			5c Does the program use terms that imply individual responsibility for health and wellbeing, such as “lifestyle” and “unhealthy choices”?						
6	Critical: Practitioner works with people as an ally	Critical: Working with people transparently as a culturally and socially sensitive and reflexive ally and resource respectful of all aspects of diversity	6a Does the practitioner privilege the diverse voices and lived experiences of priority populations?						
			6b Does the practitioner acknowledge their own privilege within the cultural and social contexts of their work?						
	*Selective: Practitioner works on people as an expert*	*Selective: Working on people as an outside expert without explicit attention to the relevant cultural and social context or all aspects of diversity*.	6c Does the practitioner consistently use allyship rather than expert oriented discourse?						
7	Critical: Empowering engagement processes	Critical: Using participatory enabling processes that empower and meaningfully engage priority populations in collaborative governance and decision making about health promotion programs designed with them	7a Are members of the priority population actively involved in the community assessment, planning, implementation, and evaluation phases of the health promotion program?						
			7b Does the program include collaborative governance and decision-making structures and processes?						
	*Selective: Disempowering engagement processes*	*Selective: Using non-participatory patriarchal processes that “target” people identified as “at risk” and limit or exclude their engagement in governance and decision making about health promotion “interventions” designed for them*	7c Is the program discourse appropriate for the priority population as opposed to being jargonistic and exclusionary?						
8	Critical: Comprehensive use of theories, models, and evidence	Critical: Basing health promotion practice on the comprehensive application of appropriate theories, models, and evidence across community assessment, planning, implementation, and evaluation components of a health promotion program to ensure sustainable health and wellbeing outcomes	8a Is the program based on a broad range of evidence types including community views, empirical studies, epidemiological data, and relevant practice-oriented theories/models?						
			8b Does the program identify specific theory and/or health promotion models or frameworks as its foundation?						
	*Selective: Limited or selective use of theory, models, and evidence*	*Selective: Basing health promotion practice on selective application of theories, models, and evidence across community assessment, planning, implementation, and/or evaluation components of a health promotion program*.	8c Are all components of the theories and models applied in the program?						
9	Critical: Maximum beneficence	Critical: Actively considering what the benefits of a health promotion program may be to the full range of beneficiaries particularly those with less structural and systemic advantage	9a Does the program identify the full range people who may benefit from the program?						
			9b Does the program prioritize strategies that benefit priority populations with less structural and systemic advantage?						
	*Selective: Limited beneficence*	*Selective: Considering what the benefits of a health promotion program may be to a limited range of beneficiaries who may have structural and systemic advantage*	9c Does the program evaluation focus on the assessment of health and wellbeing outcomes for priority populations with less structural and systemic advantage?						
10	Critical: Non-maleficence is a priority consideration	Critical: Actively considering who may be harmed by the health promotion program and in what way; taking steps to minimize or avoid this harm; and communicating the risk of harm involved in a truthful and open manner	10a Does the program explicitly identify who may be harmed by the program and in what way?						
			10b Does the program include strategies to minimize or avoid potential harms?						
	*Selective: Scope of maleficence not fully considered*	*Selective: Considering only a limited range of potential harms, in part due to a belief that health promotion programs will automatically result in positive health outcomes, and/or due to assumptions derived from structural and systemic advantage*	10c Does the program include strategies to communicate the risk of harm that may arise from the program?						

For each reflective question in QATCHEPP, users score the practice as strongly (2 points), somewhat (1 point), or minimally or not at all (0 points) aligned with critical health promotion. If there is no relevant evidence within program documents, the question is scored as 0 points. Each reflective question is of equal value with no questions weighted more heavily than any others. The scores for the three reflective questions for each value and associated principle are summed to create 10 individual scores, which are summed to create a summary score out of 60 and then converted to a percentage value. A summary score of 85% (54/60) is indicative of strongly critical health promotion practice. This requires a minimum of 24 of the 30 reflective questions to be rated as strongly critical and an additional six questions to be rated as somewhat critical. On average, this means that at least eight of the 10 values and principles would have been rated as strongly critical. Based on our academic experience, it is our subjective judgment that 85% is generally the lower boundary of the highest category of achievement. A summary score of between 50% (30/60) and <85% (53/60) is indicative of somewhat critical health promotion. This requires a minimum of 15 of the 30 reflective questions to be rated as somewhat critical, meaning that on average, at least five of the 10 values and principles would have been rated as somewhat critical. A score of below 50% is indicative of practice that minimally or does not reflect critical health promotion. Results of QATCHEPP can be analyzed and reflected on for each question, each value and principle, and overall.

## 4. Discussion

The aim of this project was to develop a quality assessment tool to support critical reflection on health promotion practice. The Quality Assessment Tool for Critical Health Promotion Practice (QATCHEPP) includes 10 values and associated principles, three reflective questions for each value and principle, and a scoring system. QATCHEPP assists practitioners to critically reflect on a health promotion program, which includes any project, policy, strategy, or initiative designed to improve health equity. The purpose of QATCHEPP is to support the reorientation of health promotion practice toward a more critical approach.

Despite health promotion's critical foundations explicated in the Ottawa Charter onwards, much health promotion activity is still selective rather than comprehensive (11, 18) or critical (15, 55). It is important therefore that tools to support researchers and practitioners to critically reflect on the normative content of policies and programs so that they align with critical health promotion are incorporated in health promotion models. The critical health promotion values and principles in the Red Lotus Critical Health Promotion Model form the basis of QATCHEPP, which can be used as part of the RLCHPM or independently as part of a quality assessment process.

Quality assessment of health promotion practice is important to ensure that health promotion programs contribute to health equity. Quality assessment tools have made an important contribution to quality improvement in health promotion practice (70). Whilst there is a range of quality assessment tools available to practitioners, they primarily focus on assessing the quality of the technical aspects of practice. For example, the HERO Health and Well-Being Best Practices Scorecard includes questions in six sections: strategic planning, organizational and cultural support, programs, program implementation, participation strategies, and measurement and evaluation (31). Likewise, Preffi 2.0 does not include any reference to the values and/or principles of health promotion (36). The Criteria for Good Practice in Health Promotion Addressing Social Determinants includes some concepts that could be regarded as values, and provides an excellent framework describing levels of implementation, however it is self-described as being focused on the technical aspects of practice (38). Another limitation of many of the quality assessment tools is the lack of a scoring system. For example, whilst the Healthy Austria Fund quality criteria include a mix of technical principles (for example networking) and conceptual health promotion principles (for example health equity), it does not include a scoring system or process for assessing the extent to which programs meet the criteria.

There has been considerable discussion within the health promotion field about the values and principles that should inform and be evident in good health promotion practice (16, 17, 71), and a set of values is included in the International Union for Health Promotion and Education's Competency Standards Framework (1). There are calls for the explicit adoption of critical reflection in health promotion practice (59, 72), but to date, critical reflection is not included in the IUHPE competency standards. Furthermore, quality assessment tools do not require critical reflection on or assessment of the values and principles that underpin the practice.

To our knowledge, QATCHEPP is the first quality assessment tool for critical health promotion that is underpinned by CSH. This theoretical underpinning provides heuristic support to enable practitioners to engage in boundary critique through critical reflection to assess the extent to which a health promotion program aligns with critical health promotion. It provides the evidence for the reorientation of programs toward a more critical approach, which is essential for addressing structural and systemic determinants of health and wellbeing to enhance health equity (2, 5, 12–14, 73). The structure of QATCHEPP is designed to enable the development of the critical reflection competence of practitioners to engage in systems thinking and reflective discourse to identify the dominant values of a health promotion program (65–67). Whilst the language used in some of the values, principles, or questions may be unfamiliar to some, it is congruent with critical theory. An essential aspect of a critical approach is to embrace uncertainty and new ways of knowing, being, and doing, which enables transformation to more critical practice.

CSH boundary critique involves using a heuristic to ask critical questions about *who/what is* and *who/what ought to be* the four sources of influences of the system, in this case the health promotion program, including sources of motivation, sources of control, sources of knowledge, and sources of legitimacy (68). For example, with respect to sources of motivation, QATCHEPP includes questions about *who is* and *ought to be* the beneficiaries of a health promotion program, and *what is* and *ought to be* the purpose of the program. With respect to sources of control, QATCHEPP includes questions about *who is* and *ought to be* the decision makers, and *what is* and *ought to be* within the scope of decision makers in a health promotion program. With respect to sources of knowledge, QATCHEPP includes questions about *what* knowledge and *who's* knowledge *is* and *ought to be* valued and *what is* and *ought to be* the role of the practitioner in a health promotion program. With respect to sources of legitimacy, QATCHEPP includes questions about *who is* and *ought to be* the priority population for a health promotion program, *what is* and *ought to be* the process for their authentic participation in all stages of program design and implementation, and *what is* and *ought to be* the strategy to minimize or avoid potential harm. Other questions within QATCHEPP further explore these sources of influence.

QATCHEPP can be used by individual or teams of practitioners to guide the design of health promotion programs or critique planned, current, or past programs. It is scalable and can be used to assess small scale health promotion programs at a local level through to national and international level program, policies, and strategies. QATCHEPP can be used by practitioners whose main role is health promotion practice within government, non-government, or community organizations. It can also be used by those for whom health promotion is a component of their role, for example clinical and allied health practitioners, educators, urban planners, climate and social justice activists, and by people in the community who may be involved in health promotion programs. Research on the application of quality assessment tools demonstrates that practitioners are reliable assessors of their own health promotion practice (74). QATCHEPP could be adopted as a quality assessment tool by funding bodies, journals, conference convenors, and ethics review boards. We now provide four examples of how QATCHEPP may be used in different health promotion practice contexts.

Using QATCHEPP, a practitioner may identify that the health promotion program they are working on is rooted in the biomedical-behavioral health paradigm, which is reflective of selective health promotion. Evidence to support this assessment is that the program goal is to reduce cardiovascular disease, and the objectives focus on reducing behavioral and physiological risk factors. The holistic health paradigm value and associated principle can then be used to invoke questions about how to reorient the program toward framing health as a complex concept that includes physical, mental, spiritual, social, cultural, and environmental aspects of wellbeing. The pilot test of QATCHEPP we conducted with students provided early evidence of its effectiveness to support reorientation of health promotion toward a more critical approach. The two graduate students who used QATCHEPP to critique the health promotion program they were working on in practice reported that it was extremely valuable for highlighting aspects of the program that had not been considered to date or could be improved. They plan to present the findings of their critical reflection to their managers, and advocate for reorientation of the program toward a more critical approach by addressing the specific aspects identified as being somewhat or minimally critical.

A community organization may use QATCHEPP to conduct an internal review of a current health promotion program. In another example, focused on increasing rates of volunteering within their community as a strategy for enhancing social health and wellbeing. They identify that the program is limited to addressing determinants of volunteering at the individual level, which is reflective of selective health promotion. Evidence to support this assessment is that the program strategies are exclusively focused on changing individual people's knowledge and attitudes about volunteering. They use the findings to invoke questions about how to reorient the program to use systems science to identify the full range of intersecting determinants of volunteering at multiple levels.

An assessor of a funding application for a new Health Promoting Schools program may use QATCHEPP to identify that the proposed program is focused on schools in middle and higher socioeconomic areas that benefit from structural and systemic privilege and power, which is reflective of selective health promotion. Evidence to support this assessment is that the priority population was determined by ease of access to these schools rather than equity considerations. In addition, the students in these schools are described as the “target group.” As a result, the assessor provides feedback to invoke questions about how to reorient the proposed program to prioritize schools in lower socioeconomic areas that are most impacted by the inequitable distribution of structural and systemic privilege and power. In addition, they suggest that the applicant reconsider their choice of language to describe the priority population.

A journal reviewer may use QATCHEPP to identify that a manuscript about a mental health promotion program does not describe any potential harms that may have arisen from the program, which is reflective of selective health promotion. Evidence to support this assessment is that the manuscript fails to address the risk that the program may have inadvertently increased stigmatization of people with mental health issues. They provide feedback to invoke questions about how the authors can revise the manuscript to include information about who may have been harmed by the program and in what way, what steps were taken to minimize or avoid this harm, and how the risk of harm was communicated.

These are just some examples of how QATCHEPP may be used to enhance critical practice. Although the results of the pilot test are promising, QATCHEPP still needs to be tested in a broad range of professional contexts. To support practitioners' use, we plan to develop a digital platform for QATCHEPP with hyperlinks to a user guide that includes more detailed explanations of the values and principles, reflective questions, and types of evidence for each response category and score. We also intend to develop a series of publicly available videos providing guidance for QATCHEPP users. These strategies will contribute to enhancing the utility and reliability of the tool. Further research is required to evaluate the application of QATCHEPP in a full range of practice environments and determine the intra-rater, inter-rater, and test-rest reliability of QATCHEPP as a quality assessment tool.

A strength of QATCHEPP is that it addresses the need for a quality assessment tool focused specifically on critical health promotion. To our knowledge, it is the only tool to do so. Due to its critical theoretical foundation, it extends existing quality assessment tools beyond technical aspects of health promotion practice to incorporate underlying values and principles of a critical approach. QATCHEPP can be used in a broad range of health promotion contexts, by a variety of users, for multiple purposes. QATCHEPP is the result of over 15 years of systematic, continuous refinement of the Red Lotus Critical Health Promotion Model in response to user reflection and feedback. The reflective questions were developed by us as researchers with extensive practice and academic experience in a critical health promotion approach. As such, the questions reflect our beliefs about the intent of the values and principles and how each is and ought to be operationalized in practice. Consistent with the constructionist epistemology, we acknowledge that other researchers may have different beliefs about what critical health promotion is and ought to be. Similarly, users of QATCHEPP will interpret the values and principles and reflective questions based on their own practice experience. As such, the evidence they identify to support their assessment for each question will likewise be informed by their own professional lived experience.

A limitation in the interpretation of the results of QATCHEPP is the arbitrary scoring for individual reflective questions, the summary score, and the cut off points for the overall assessment of the program as strongly, somewhat, or minimally critical health promotion. These numerical and categorical results generated by QATCHEPP are intended to provide a guide to inform quality improvement of programs rather than a summative judgement. A further limitation is that QATCHEPP has only been tested informally and with graduate students. Further research is required to determine the reliability of the instrument in a range of practice contexts.

## 5. Conclusion

The purpose of health promotion practice is to develop, implement, and evaluate health promotion programs to bring about changes in the determinants of health and wellbeing to enhance health equity. QATCHEPP provides heuristic support for practitioners to engage in critical reflection to assess the extent to which a health promotion program aligns with critical health promotion. QATCHEPP can be used as part of the Red Lotus Critical Promotion Model or as an independent quality assessment tool to support the orientation of health promotion programs toward critical health promotion practice. This is essential to ensure that health promotion practice contributes to enhancing health equity.

## Data availability statement

The original contributions presented in the study are included in the article, further inquiries can be directed to the corresponding author.

## Author contributions

LO'H and JT: conceptualization, methodology, data analysis, writing—original draft preparation, and writing—reviewing and editing. All authors contributed to the article and approved the submitted version.
